# Annotations

**Published:** 1897-11-20

**Authors:** 


### ANNOTATIONS.

### Inspection of Frozen Meat.

The annual report of Dr. Collingridge, medical officer of health to the Port of London, contains, among other interesting matter, some important remarks upon the extent of the frozen meat trade and on the inspection to which this form of food is subjected. At the present time there are 123 vessels engaged in bringing frozen meat to London, and these vessels have among them a total carrying capacity of 4,529,400 carcases. The great majority of these carcases come from New Zealand and Australia, the rest from the River Plate. While these vessels are discharging frequent visits are paid to them to ascertain if their cargoes are in good condition, and if any damaged meat is met with then the supervision is at once made more strict, so as often actually to mean that every carcase is individually inspected. Some of the meat is sent away by rail, some is discharged into craft for conveyance to refrigerators up the river, and some is landed for storage at the docks. No examination in the stores has been found to be either practicable or desirable, and at none of the meat stores in London is there any inspection of the meat on leaving. Dr. Collingridge gives figures to show that a complete inspection of each carcase on leaving the refrigerator would entail a large increase of the staff employed, would seriously interfere with trade, and would not lead to the detection of any but a small quantity of unsound meat. This may all be quite true, nevertheless the fact remains that all this immense mass of frozen meat is constantly poured into the London market without any further inspection than what is necessary to detect what goes by the name of "unsoundness." Now this may be right or it may be wrong—the subject is too large to argue just now; but this at least is quite clear, that whenever the London County Council makes up its mind to seriously tackle the question of the slaughter-houses it will have at the same time to tackle the question of the frozen meat. It will never do to go to the expense of municipal abattoirs (which would be done chiefly for the sake of efficient inspection of English meat), and then allow London to be made the dumping-ground of all the doubtful meat of foreign countries by allowing anything to come in without inspection so long as it was frozen.

### The Physiology of Hypnotism.

There are certain limits beyond which even the modern physiologist finds it impossible to pass. In some way mind is correlated with the existence of special living structure, and in some way the functional attributes of nervous tissues are transmuted in the crucible of consciousness into the various moods of sensation, thought, and volition; but the rationale of such transformation is as inexplicable as that of the origin of matter or the commencement of life. Nevertheless, it does seem clear that the presence of the mental state may be taken as a sign of the full development of certain physiological processes; and the very fact that in the hypnotic condition certain apparently complete "mental" manifestations may take place in the equally apparent absence of consciousness and volition, make the study of the hypnotic condition one of great physiological interest. The great question may broadly be put thus: How can the fixing of attention result in such a severance of between action and volition, as we see to be the case in the hypnotic state? In a most interesting article in *Science Progress*, Professor Francis Gotch attacks this question from the physiological side, and shows how greatly modern views as to the construction of the nervous system assist us in forming a mental picture of the processes involved. The essence of these modern views lies in the non-continuity of nervous tissue. There are gaps between the different neurons, and the direction in which nervous impulses flow depends largely upon the ever-varying resistance which is offered by these gaps. That does not, perhaps, take us far; but if we recognise that all our activities are the result of a form of reflex action, inhibited or accelerated by impulses from that portion of the nervous system which are more specially connected with the consciousness, and that the nerve processes whence the impulses by which this inhibition is maintained must "jump the gap" are capable of fatigue, we seem to see, in a fashion, how it is possible, as the result of strained attention, to weary out the link with consciousness, and leave the body an automaton, subject to the uncontrolled dominion of reflex action and suggestion. This explanation is one of the very greatest interest, and all the more so from the fact that it seems to explain on the one hand how by repetition self-control may come to be the dominant factor in a man's actions, and how, in another case, by the repeated abolition of the control of the higher centres, such as occurs in those who are again and again subjected to hypnotic influence, it may no longer be necessary to fatigue the will by concentrated attention in order to produce the hypnotic state. By mere repetition a state may be induced in which the subject may become hypnotic on a gesture, on a look, or even on the mere casual occurrence of an event which has been usually connected with the onset of the trance. Of this the following curious example is given. A patient in the Salpétrière used to be hypnotised at a certain hour. One day she committed a petty theft, stealing some valuables out of a drawer which was not her own. While in the act the hour struck at which she usually was hypnotised, with the result that she fell a prey to the suggestion started by the sound of the clock, and passed at once into the accustomed cataleptic condition. There she was found red-handed, standing a stricken thief.

### The Lamp Accident Season.

An inquest was held last week on a young woman who died in St. Thomas's Hospital from burns resulting from the explosion of a paraffin lamp. It was the old tale. She blew down the chimney, and the reservoir exploded. The evidence of the lamp inspector was again the old tale—a glass container, a large air space filled with vapour, a direct communication between this explosive chamber and the flame, and nothing but a puff of air required to bring on the catastrophe. It is easy to see where the error arose, and it is easy to say that, in these days of education, people should not buy such lamps, and should not blow down the chimneys. But when we hear that during the twelvemonth ending last March forty-five lives were lost in London from lamp accidents, we cannot but feel that if a little of the legislative zeal which overflows to such small purpose at Westminster could be devoted to the prohibition of the sale of dangerous lamps, much good might be done.

